# Enhancing care in the initiation and management of insulin in older people with diabetes: A collaborative journey with older individuals and their caregivers using Experience-Based Co-Design

**DOI:** 10.1371/journal.pone.0302516

**Published:** 2024-07-29

**Authors:** Chaya Langerman, Angus Forbes, Glenn Robert

**Affiliations:** Florence Nightingale Faculty of Nursing, Midwifery and Palliative Care, King’s College London, London, United Kingdom; Universiti Kebangsaan Malaysia Faculty of Medicine: Hospital Canselor Tuanku Muhriz UKM, MALAYSIA

## Abstract

**Introduction:**

Initiating insulin therapy in older individuals with type 2 diabetes (T2DM) poses unique challenges and requires a nuanced understanding of the age-related factors that impact safety and efficacy. This study employed Experience-Based Co-Design (EBCD) to enhance the insulin initiation and management experience for this population, emphasising a collaborative approach involving patients, caregivers, and healthcare professionals.

**Aim:**

The primary aim of the research was to develop a tailored care pathway, utilising co-design and the Behaviour Change Wheel (BCW), which addressed issues specific to older adults on insulin therapy. The study sought to identify key challenges, propose practical interventions, and construct a logic model illustrating a pathway for enhanced insulin treatment experiences.

**Methods:**

An adapted EBCD process was used which integrated the Medical Research Council (MRC) Framework and BCW. The study involved thematic synthesis, video interviews, and feedback focus groups with patients, caregivers, and healthcare professionals. The ’Crazy Eights’ brainstorming method, as part of the co-design workshop, generated practical solutions which informed subsequent logic model development.

**Results:**

Focus group findings revealed distressing insulin initiation experiences, inconsistent dietary advice, and perceived disparities in care between type 1 and type 2 diabetes. The co-design workshop identified eight key challenges, leading to proposed interventions aligned with the BCW. The logic model illustrates a pathway for older individuals undergoing insulin treatment, emphasising behaviour change among patients, caregivers, and healthcare professionals.

**Conclusion:**

The collaborative efforts of participants contributed valuable insights in terms of the unique educational and emotional needs of patients, the importance of care continuity and of improving access to specialist services. Findings from this study can be used to inform and enhance tailored support strategies for older adults with T2DM during their insulin transition and ongoing management.

## Introduction

Insulin initiation in older people with T2DM is an important step in achieving optimal glycaemic control and preventing diabetes-related complications [1]. As diabetes progresses, many older adults require insulin therapy to effectively manage their blood glucose levels. However, when initiating insulin in this population it is important to consider age-related factors that can impact on the safety and efficacy of the therapy. These factors can include increased frailty, cognitive decline and functional impairment [2]. Previous studies have shown that older adults with T2DM may have multiple challenges to insulin initiation [3–5]. These include a fear of injections, worries about hypoglycaemia, and concerns about potential medication side effects [6, 7]. Healthcare providers have been urged to address these issues through patient education, improved communication, and reassurance about the benefits of insulin therapy [6, 8].

A recent thematic synthesis focused on the issues experienced by older people with T2DM who were using insulin [9]. It found that older adults often harbour negative attitudes towards transitioning to insulin and that changes to their cognitive and physical functioning could impact their ability to use insulin appropriately. Additionally, it highlighted the importance of considering carers’ needs, given the wider physical, emotional, and financial consequences of diabetes that can affect families [9]. Such findings identify a need for an approach to insulin management which is tailored for older people and their carers. The importance of involving vulnerable individuals in research to enhance services has been acknowledged [10]. However, few studies have involved service users and professionals to collaboratively develop effective care for older people with diabetes. In this paper, we describe a co-design study involving older people, their carers and health professionals. The aim of the study was to understand how best to organise services and enable professionals to effectively support older people transitioning to insulin.

Co-design is a creative way of understanding experiences and improving services through the adoption of ‘human-centred’ design methods, tools, and processes [11]. Despite variations in co-design approaches, all emphasise sharing power with stakeholders to make informed design decisions and develop more acceptable and feasible interventions [12, 13]. One method of co-design which was developed to improve the experiences of patients in healthcare services is EBCD [14, 15]. Developed in 2005, EBCD has been proven as an effective way of improving healthcare services through participatory action research involving patients, families, and staff [16–19]. As a result of using the EBCD approach [20], a number of service improvements activities have been reported [21]. For example, in one recent study [17], older individuals experiencing frailty and their carers informed the optimisation of a multifaceted intervention to improve the process of discontinuing medications.

The approach adopted for this study was an adapted form of EBCD which focused on the perspectives of both older people with T2DM, carers and staff. It also recognised the transformative possibilities of engaging each in co-design which could lead to broader impacts on health and well-being [14, 15]. We aimed to identify the common issues that older people face in relation to their use of insulin, and work collaboratively with all stakeholders to identify supportive interventions and solutions [22, 23]. It is important that new interventions which result from EBCD are underpinned by theory. During the early stages of intervention development, research focuses on how an intervention can change behaviour. We therefore utilised the Behavioural Change Wheel (BCW) [25] and its associated Behaviour Change Theory (BCT) v1 [24] as it provides a framework for the systematic design and development of behaviour change interventions. As a final step, our study sought to develop a logic model which would illustrate how novel and ongoing care procedures could be optimised for older adults with T2DM. The model was constructed to visually represent proposed service developments, professional inputs and educational interventions in terms of their delivery mechanisms, intended effects and outcomes for older people [25, 26].

## Methods

### Theoretical frameworks

Incorporating the phases of the EBCD approach as outlined by Robert et al. [14] into the MRC Framework for complex interventions [27] can enhance the engagement of both service recipients and providers. The MRC Framework comprises four broad phases of intervention identification/development, feasibility, evaluation, and implementation. EBCD fits within the development phase of the MRC framework, focusing on understanding the problem, identifying issues, and planning changes [14, 15, 28]. Patients, caregivers, and clinicians are engaged as collaborative partners throughout these different elements of an intervention’s co-design process.

EBCD is usually a six-stage process [20] which involves: 1) project set up; 2) gathering the experiences of staff; 3) filming in-depth interviews with patients and carers and/or observation, and using these to construct a short “trigger film” of patient narratives; 4) bringing patients, carers and staff together to view the trigger film and to identify shared priorities for change; 5) working in small project groups of patients and staff to focus on challenges prioritised in previous workshops to find solutions; and 6) holding an event attended by all to celebrate the achievements of the project groups. In particular the trigger film features a range of “touchpoints,” and has been found to be a useful way to highlight patient perspectives to engage staff and patients to focus on priorities for change [29]. Touchpoints are specific moments in the care journey that elicit strong emotional feelings and play a significant role in shaping a person’s experiences [30].

The EBCD [15] approach implemented in this study was subject to certain modifications to better fit the needs of our participants and organisational context [11]. These modifications were in response to the challenges of recruiting and retaining older participants across multiple events, as many were burdened by serious health conditions. Thus, the EBCD process was shortened to include just a single co-design meeting and omitted the smaller co-design project groups and final celebration event. Although local adaptations to the EBCD approach are common [22, 31], their effect on our research data is further considered under the limitations section.

Improving the experience of older people on insulin may depend on changing the behaviour of healthcare professionals and of the older people themselves. Recent approaches have integrated EBCD with the Behaviour Change Wheel [32] along with its associated Behaviour Change Theory (BCT) v1 [24] to guide the co-design of complex interventions which involve service users and various stakeholders [33]. The BCW is a comprehensive framework that aligns behaviour change outcomes with intervention elements. It was developed from a systematic review of 19 behaviour change frameworks used in previous interventions [25], offering a structured approach to understanding and implementing effective behaviour change strategies. The BCW has been applied in interventions aimed at fostering behaviour change in both individuals diagnosed with diabetes and healthcare professionals [26]. The BCW [32] along with its associated BCTv1 [24] features nine intervention functions (i.e., ways in which an intervention might change behaviour). These are: education, persuasion, incentivisation, coercion, training, enablement, environmental restructuring and restrictions. Each of these functions has the potential to influence behaviour change. The BCW is therefore a useful model for shaping the ‘active ingredients’ of interventions, and has been used in a previous study which developed behavioural interventions for older people [34].

**Fig 1:**
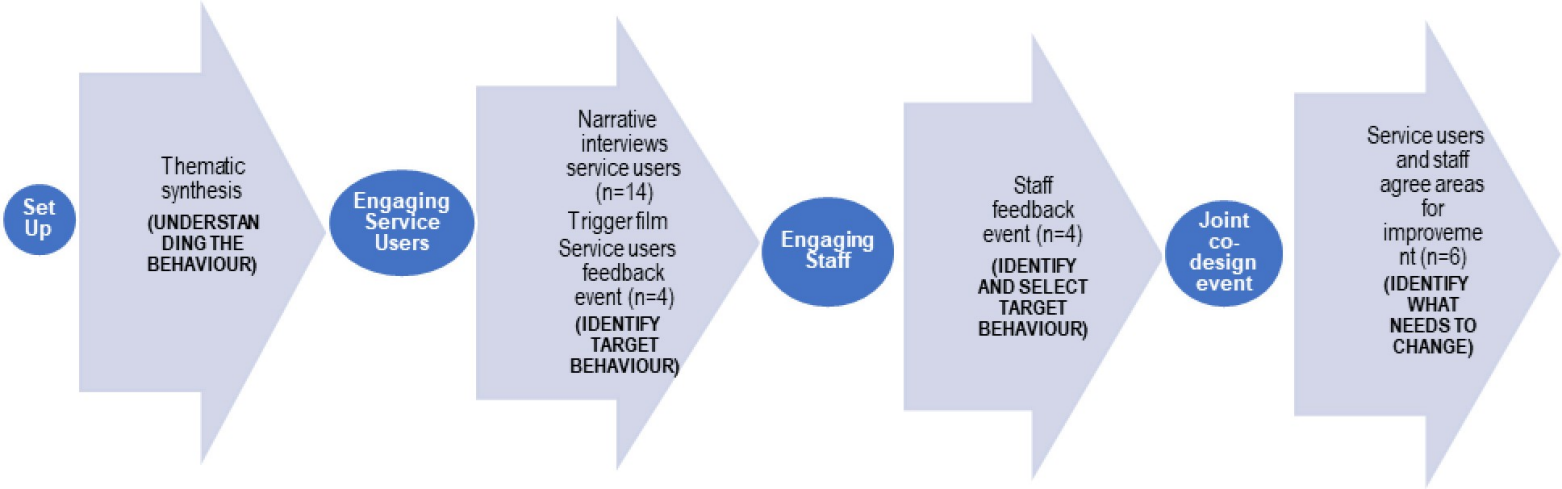
Integrated co-design behaviour change model.

In this study, we integrated the EBCD approach with the initial stage of the BCW. This stage encompasses understanding the behaviour through: thematic synthesis of relevant literature to identify common themes (stage 1 of EBCD); identifying and selecting the target behaviour by conducting interviews (stage 2 of EBCD) followed by feedback workshops with patients, caregivers, and healthcare professionals (HCPs); selecting the target behaviour (stages 2 and 3 of EBCD) and determining what aspects need to change at the co-design focus group (stage 4 of EBCD). Fig 1 provides a visual representation of this process.

### Procedure

#### Stage 1: Setting up the project

Initially a thematic synthesis of the experiences of older people using insulin was performed [9]. Qualitative video interviews were then undertaken with 14 older people and their carers to gather experiential data on their perspectives on insulin use, and also to construct a trigger film featuring emotional touchpoints. Using purposive sampling, we then recruited older people and informal carers to take part in the co-design activities. The inclusion criteria were: 1) potential participants who were over 70, diagnosed with T2DM and who had been undergoing insulin treatment for between 6 to 48 months; 2) informal carers (friends or family members) caring for this population. Carers could be those supporting participating older people, but their relatives did not have to be taking part for them to be eligible; and 3) diabetes specialist nurses who were affiliated with King’s College London and specialised in the management of diabetes in older individuals on insulin. In accordance with the online EBCD toolkit [35] which provides guidance on how to conduct EBCD research, we aimed to recruit up to 15 patients, carers and staff participants for stages 2–4 of the study. We selected individuals aged over 70 years, aligning with the European Diabetes Working Party’s definition of older people with T2DM [36]. The window of insulin initiation of between 6–48 months was adopted to ensure people were familiar with the regimen but to avoid difficulty with longer term recall of transition experiences.

#### Recruitment

Due to COVID-19 restrictions at the start of the study, all participants were recruited virtually from across the UK. Recruitment took place through the websites of the voluntary organisation Diabetes UK (DUK) and from the research networks of the National Institute of Health Research (NIHR). These platforms served as online spaces for hosting the study advertisement (see S1 File). Interested participants could directly email the lead researcher (CL) if they wished to participate and received written information about the various focus groups and workshops. They were also able to talk to the lead researcher about the purpose of the research and implications of participating. People were invited to attend all the activities in the EBCD process but were also offered the flexibility to choose which ones they wished to participate in. Recruitment commenced on April 1, 2022, and concluded on May 25, 2023.

The lead researcher, CL, is an academic researcher with an interest in co-design and peoples’ experiences of healthcare including diabetes services. Other team members were GR, who is an academic methodological expert in EBCD, and AF who is also an academic and specialist clinical nurse with experience of treating people with diabetes within the UK’s National Health Service (NHS).

#### Ethics

Approval for the study’s ethical aspects was secured from the Psychiatry, Nursing and Midwifery Research Ethics Subcommittee at King’s College London, UK (references LRS/DP-21/22-27077 and RESCM-22/23-27077). All participants provided their consent in writing before taking part, including explicit permission for excerpts of their video interview to feature on the trigger film. To maintain confidentiality, we ensured that the interviewer was working in a private space at the time of the interview recording and that the interviewee was also alone unless they specifically wished their carer to be present.

### Stage 2: Development of trigger film from interviews with older people and carers

#### Interview analysis

The interviews were recorded digitally, transcribed word-for-word, and then loaded into NVivo version 10. Framework analysis was adopted as the analytical method because of its adaptable but well-structured approach, and lack of strict alignment with a specific theoretical framework [37]. Trustworthiness [38] was ensured through employing a robust team-based approach to analysis, decisions being documented to ensure transparency, audit trails recorded, and reflexivity encouraged. Three primary themes were generated from the interview analysis, each consisting of six sub-themes: Theme 1 –The transition to insulin (subthemes: adapting to insulin use, negative emotions connected with insulin use); Theme 2 –What we need from a service (subthemes: better information about insulin, a holistic streamlined service); and Theme 3 –Empowering older people (subthemes: supporting autonomy, do we matter?). Detailed findings from these interviews can be found in a separate paper (*publication in process*).

#### Trigger film development

In the next stage, researchers CL and GR repeatedly watched the raw footage of the videoed interviews to select touchpoint moments and create a trigger film [19]. Guided by the themes generated by the qualitative analysis, the researchers looked for key interaction moments between older people and services where something could have been done better or revealed a particularly good experience [39]. These touchpoints were then classified into one of five film chapters which broadly reflected the chronological stages of transitioning to insulin (see Table 1). Initially a 60-minute film was created by CL and GR, and this was subsequently refined by the same authors to create a cohesive and insightful 30-minute film. Support from a professional film producer, and the use of headings and subtitles ensured that the film was of good quality before viewing by stakeholders. Identified touchpoints included: insufficient provision of insulin-related information; the capacity for self-administration of insulin; restricted access to and communication with healthcare experts; and emotional well-being concerns.

**Table 1:** Trigger film chapters and related touchpoints.

Film Chapter	Touchpoints
Referral and starting insulin	1. Lack of integrated and uniformed diabetes care provision across primary and secondary care
	2. Problematic and negative communication of the need for insulin
	3. Lack of individualised approach in insulin initiation
Insulin	4. Lack of confidence in handling insulin
	5. Patients aspire to assume greater authority over their own bodies and have more say in the way their health is managed.
Information	6. Lack of individualised information reflecting variability in learning styles, cognitive ability, cultural background, and information needs.
Diet	7. Lack of diet and lifestyle advice in relation to insulin.
Ongoing management	8. Lack of access and continuity to general practitioner (GP)

### Stage 3: Patient/carer and Healthcare professionals (HCPs) feedback focus groups

In the third stage, two online focus groups were organised to gather feedback on the trigger film from both patients and their caregivers. The groups lasted between 1–2 hours and were audio-recorded with permission. They were facilitated by CL who has extensive experience of running group discussions within research settings. Patient/carer groups were held virtually on Microsoft (MS) Teams due to patient preferences. Participants were prepared and guided on the use of technology by the researcher. A third focus group was held with clinicians based at King’s College London to gather staff feedback on the trigger film. This was a hybrid focus group (in person and virtual) due to staff preference. The hybrid focus group was conducted with staff present in a room equipped with a TV screen displaying those staff attending virtually. All participants were briefed about the format, and most were familiar with this way of working.

The trigger film was viewed one chapter at a time and participants were asked to validate that the issues identified in the film resonated with them. In all three focus groups, participants were also invited to identify any significant issues that they felt had not been represented. Participants were then encouraged to think about what collaborative efforts could be undertaken by patients, carers, and healthcare staff to improve each issue identified. Participants were urged to draw from their personal experiences when considering these improvements but also to consider the wider impact of such changes. While participants were free to engage in open discussions among themselves, CL facilitated these dialogues by guiding conversations back to insulin initiation and ongoing management when necessary. The results of these focus groups are documented in Table 3.

### Stage 4: Joint co-design workshop

Patients, carers and HCPs from the previous focus groups were subsequently invited to a joint workshop. Due to participant preference, this workshop was conducted remotely on Microsoft (MS) Teams. All participants collectively reviewed the challenges identified during the previous focus groups and decided jointly which were most important for improving care. Participants were then tasked with generating ‘How might we?’ (HMW) statements. HMW is a design thinking method [40] which involves defining a problem, an action to address the problem, and then considering the likely impact of this action. Brainstorming was then encouraged through the use of a ‘Crazy Eights’ technique [41, 42] to develop novel solutions to these problems. This approach asks participants to generate as many ideas or solutions as possible within an eight-minute timeframe to encourage creativity and avoid overthinking. Finally, participants shared their ideas from the brainstorming session and voted on which held most promise as a potential intervention to improve diabetes services.

Stages 2 to 4 took approximately 30 months to complete due to disruptions to the research process caused by COVID-19 restrictions. Given social contact restrictions and the difficulties retaining older participants across multiple phases of the research, only one co-design workshop was conducted, and no final celebration event was held.

#### Development of the logic model

After the co-design events, the research aimed to develop a logic model to visually represent possible interventions which targeted behaviour to improve care outcomes for older people transitioning and managing insulin treatment. We chose to develop a basic Type 1 model [26] aligned with the Kellogg Foundation’s approach [43]. This model provides a foundational framework for intervention planning and is considered resource efficient during the early planning phase of an intervention.

The logic model was developed after synthesising feedback from the co-design discussions, the thematic synthesis and the interview data. The interview data themes, and associated trigger film were an important catalyst to reaching consensus about what challenges should be prioritised. This information therefore formed the basis of the logic model. Most logic models are usually structured around a series of causal relationships, often articulated through “If-Then” statements that guide the flow of activities, outputs, and outcomes of logic models [44]. In this research, the process of forming “If-Then” statements was carried out by the researcher after the co-design workshop had been conducted. The statements were based closely on discussion between the participants and the researcher (CL) at the workshop. The solutions identified to address challenges formed the “If” part of the statements. The “Then” parts reflected participants’ perceptions of the impact from these changes. Initially the data extracted from the co-design meeting was organised into distinct ’If-Then’’ tables for each topic. Subsequently, these tables were amalgamated to create comprehensive “If-Then’’ statements that underpinned the program’s logic. Evidence from the thematic synthesis and wider literature was also used to generate outcomes within the model. The initial model underwent further refinement through sharing it with healthcare professionals and researchers to ensure feasibility and accuracy, particularly in terms of the potential short and longer-term outcomes.

The individual ideas emerging from the Crazy Eights sessions were not included in the logic model as they would require further development. However, intervention categories e.g. ‘positive conversations’ were generated for the logic model to indicate the broad areas where intervention development was needed. Accordingly, the Crazy Eights suggestions were classified in terms of their intervention function using the BCW in order to aid future selection and development of these interventions.

The study reporting adhered to the COREQ guidelines [45].

## Results

Overall, 18 participants engaged in the EBCD process across all phases. Two patients and one carer participated in all stages (see Table 2).

**Table 2:** Characteristics of EBCD participants.

Study identifier	Gender	Age	Participant type	Duration on insulin (months)	EBCD activity
					(I = interview, TF = Trigger film, F = feedback, CD = co-design)
NO1	Male	71	patient	24	I, TF
NO2	Male	73	patient	6	I, TF, F, CD
NO3	Male	71	patient	36	I, TF
NO4	Female	73	patient	30	I, TF, F
NO5	Female	-	carer	NA	I, TF
NO6	Male	75	patient	6	I, TF
NO7	Male	72	patient	24	I, TF, CD
NO8	Male	76	patient	12	I, TF
NO9	Female	-	carer	NA	I, TF
NO10	Male	71	patient	9	I, TF, F, CD
NO11	Female	-	carer	NA	I, TF
NO12	Female	76	patient	40	I, TF
NO13	Female	-	Carer	NA	I, TF, F, CD
NO14	Female	74	patient	24	I, TF
NO15	Female	-	Nurse	NA	F, CD
NO16	Female	-	Nurse	NA	F
NO17	Female	-	Nurse	NA	F
NO18	Male	-	Nurse	NA	F, CD

### Findings from older people/carer and HCP focus groups

Stakeholders responded to the issues featured in each chapter of the trigger film (Table 3). Older people recognised the negative feelings experienced during insulin initiation. They also shared their perspectives on inconsistent dietary advice, the disconnection between primary and secondary care and limited access to personalised information. Furthermore, older people noted a perceived disparity in the care provided for type 1 and type 2 diabetes, underscoring the importance of greater involvement of patients and caregivers in decision-making. HCPs agreed with the challenges expressed by older people and their caregivers.

**Table 3:** Summary of key challenges identified in the feedback focus groups and concordance between groups.

Film chapter	Challenges as perceived by patients/carers	Perceptions of HCPs
Referral and starting insulin.	Transitioning to insulin can be traumatic and cause anxiety and confusion.	HCPs acknowledged that older people could react very negatively when they are told about the need to transition to insulin.
	*‘It’s a grieving process in a way*. *When you get diagnosed with something and then you have to come to terms with it and learn how to work with it yourself*, *and you know you’re the best judge of how you feel*, *but you need the expertise and support initially and maybe you know concurrent’ (carer*, *N013)*	*‘You get into insulin and that*, *you know*, *almost like death is the next point*. *And I guess that needs a bit more unpacking*, *doesn’t it*?*’ (HCP*, *N016)*
	People found that Information was not tailored for older adults and was too general for people’s needs.	HCP agreed that the information resources available for older people were limited.
	*‘It’s just like the finger*. *I’m relate to it because as I said earlier*, *everybody’s different*, *you know*, *just like my finger everybody*, *they’re never the same’*. *(patient*, *N02)*	‘*And I think that’s key and it like*.* *.* *.*you know we assume a standard leaflet works for everybody*. *It might not do and it’s just checking on that and the inconsistency of information that people are provided*.*’ (HCP*, *N018)*
Insulin experiences	People feel that they are not provided with sufficient kits to test their blood glucose.	No perspective expressed by HCP
	*‘Initially you know I have problem with my GP because I didn’t have enough blood testing strips*, *he said*. *He was coming through*. *It’s coming through his budget*, *his surgery budget*, *so I had to convince him’ (patient*, *N02)*	
	No perspectives from older people/carers.	HCPs considered that carers are important to successful insulin management for some older people but acknowledged that they may not all wish to fulfil that role.
		HCPs spoke about how it was key to establish appropriate outcomes for older people to help them engage with treatment.
		‘*Masses of frustration like resentments*, *all sorts of difficulties within relationships which were new since the children or their siblings*, *or the usually the spouse*, *has to step up into this more caring role’*. *So*, *carers are a big part of*, *you know*, *their (e*.*g*. *patients) life with diabetes and actually have a bigger Influence over their outcomes probably than anyone else*.*’ (HCP*, *N016)*
		*‘I think we need to establish the need for insulin very carefully in the older person*, *if the patient is not convinced–- it’s a lost case*. *So*, *i’’s not what we think the person would benefit from*. *I’’s what they (e*.*g*., *patients) think you know*, *we can sell them the benefits (for example long term protection of eyes will not work on older)–- Tell a 75–80-year-old this is going to protect your eyes in the next 20 years*, *that might not be the thing that the’’re going to take away*.*’ (HCP*, *N017)*
		*‘Having more energy*, *you know*, *almost*, *sell it*, *be honest about what the benefits are and what the negatives are as well*. *That could be really important in someone wh’’s old and feel tired and has incontinence*.*’ (HCP*, *N017)*
Information	Older people sometimes found it a challenge to understand, remember and access information related to their diabetes.	HCPs acknowledged that retaining information could be difficult for older people and more support and educational opportunities are needed.
	*‘Again*, *it is all about understanding and if people knew that*, *maybe they would’’t feel so scared because there could be trying to do that*. *And if there was some kind of course that people could go on*, *meet each other*, *and maybe tha’’s something that could happen through GP surgeries or elsewhere once a year*, *I think that when people meet others that ‘’ve got the same problem*.*’ (carer*, *N013)*	
		*‘So as a healthcare professional*, *we think w’’ve explained it all*, *but i’’s that accessible to people who are sitting in front of us and that is one thing we probably need to think about like carefully*.*’ (HCP*, *N018)*
		*‘Good point about reinforcing information*. *HCPs should reinforce information to patients over and over again–- I’’s called the Forgotten…i’’s not just a course*.*’ (HCP*, *N015)*
Diet	Older people wanted more dietary and lifestyle advice from specialists in relation to insulin use.	HCPs agreed that non-specialised staff sometimes provide inconsistent or insufficiently detailed dietary advice.
	*‘I did request that my GP to send me back to a dietitian*. *He said there is no point and he gave me a list of websites*. *I requested because I wanted to review my diet and then see how I can manage it*. *But he (GP) said no*, *that it was not necessary*. *I do’’t know how much that will cost the surgery’*. *(patient*, *N03)*	*‘w’’ve probably in the past of hammered a lot of diabetes diet kind of thing*, *which do’’t really necessarily need to be*, *I think a general healthy diet*.*’ (HCP*, *N017)*
Ongoing management	Older people reported a lack of access to general practitioner and poor continuity of care.	HCPs were aware of poorly integrated and uniformed diabetes care provision across primary and secondary care.
	*‘So if you phone up*, *you could get a different doctor every time you phone up and another*, *they can have access to your records*, *but they do’’t personally know what yo’’ve been talking about previously*. *So ther’’s lack of continuity of consultation’*. *(patient*, *N010)*	*‘Some areas are hospital-based care…the medical complex people… we refer them and even then …short of time as possible they (hospital staff) see them (the patients) and then they (hospital staff) refer them (patients) back (to primary care) and often blame the patients’ (HCP*, *N017)*
	*‘I used to go and see the doctor*..*the same doctor*, *every time*, *but nowadays i’’s never happens like that*. *Every time you go*, *you see somebody else*. *So you*, *you start all over again*. *It is difficult for me*. *I wish I was assigned one doctor every time*, *same doctor*.*’ (patient*, *N08)*	
	No perspectives from older people/carers.	HCPs noted some older people lack confidence in handling insulin and adjusting their dosage
		*‘Did any of them adjust the insulin rather than think if they were having hypos in the night*? *could they not have reduced their dose or did they feel they needed to have permission for it*.*’ (HCP*, *N016)*
		*‘Defining the need for insulin with people*, *older people discussing those benefits and disadvantages and*, *you know*, *having a partnership relationship*.*’ (HCP*, *N017)*
	No perspectives from older people/carers.	HCPs were aware that older people’s attitudes towards managing their health changed over time.
		*‘Establishing preference for someone to continue insulin and ask if they want to continue or change the type of insulin–- that needs to be asked at least yearly*.*’ (HCP*, *N015)*
		*‘Some people actually prefer to be told how to adjust their insulin and in an understandable way*.*’ (HCP*, *N016)*
	Older people perceived inequality between type 1 and type 2 diabetes patients in terms of expenditure and technology.	HCPs acknowledged current inequality but also that policy was changing in terms of access to technology.
	*‘So that is actually a lack of coordination in dealing with diabetes*, *I think there is a gap between Type one and Type 2’ (patient*, *N08)*	*‘They want changes*, *so we are giving flash to Type 2 now*. *Yes*, *it is in the NICE guidelines*. *I’’s just taking a while to get rolled out now*.*’ (HCP*, *N017)*
	Older people wanted to be consulted about how their care was managed.	HCPs were aware that some people were not adequately involved in decisions about their health.
	*‘The main thing is you gotta be in control of your body and to understand your own body*. *Because I believe the best doctor for you is yourself*.*’ (patient*, *N01)*	
		*‘Agreed care plan*, *is’’t it having a partnership agreement*? *does’’t seem to be*, *everyon’’s experience*, *not all HCPs doing it*.*’ (HCP*, *N015)*

### Findings from the Co-design workshop

At the beginning of the workshop, participants worked towards a final consensus on eight key challenges which would be important to focus on in future intervention development. These are presented in Table 4, column 1. Using the HMW approach, patients and staff thought of various practical ways of addressing these issues by thinking about service improvements and novel initiatives to support their care (see Table 4, column 2). Some ideas involved making changes at a service level, for example, being given access to specialist dieticians. There were also calls for better visual representation and translation of information to accommodate diverse learning styles. In addition, patients wanted more support with their blood glucose monitoring and insulin management. Participants were also asked to think about the practical impact of introducing these changes to care (Table 4, column 3). For example, participants believed that being given more self-management support would help to improve their confidence and their ability at optimising their medication use. The ‘‘Crazy Eights’’ method had generated many solutions for the ‘How might we?’ statements. For instance, to address the issue of diverse learning styles, creative suggestions included a website with translation options and auditory features akin to platforms like Audible. After this process, participants in the workshop employed a voting process to help determine which of the “Crazy Eights” ideas were most likely to bring about positive change and enhance their experiences (see Table 4 column 4).

**Table 4:** Summary of main priorities identified during co-design workshop.

Key challenges/priorities	Proposed improvement by patients, carers and HCPs (IF)	Potential impact/outcomes. (THEN)	Practical examples from co-design workshop (selected ideas from Crazy Eights)	Researcher identified BCW intervention functions
Lack of integrated and uniformed diabetes care provision across primary and secondary care (Healthcare services)	Self-help resources and E-learning that is easy to access for patients and carers to provide more flexible access to consistent information.	Older people have the ability to review blood test results and adjust insulin doses, ultimately leading to a reduction in diabetes-related complications.	Streamlining communication between hospitals and general practitioners through the use of an electronic ‘insulin passport.’	Environmental restructuring (Changing physical or social Context)
Problematic and negative communication of the need for insulin (Transition to insulin)	Initiate group sessions with other people that need to go on insulin, providing more time with clinician to digest the need for insulin, its benefits and challenges	Older people can share concerns with peers, validating their feelings and diminishing the sense of isolation, along with reducing self-blame for their condition.	Use peer groups to support patients and carers through sharing best practices with their insulin	Modelling (providing an example for people to aspire to or imitate)
Lack of individualised approach in insulin initiation (Healthcare services)	An individualised care plan adapted to the needs of the patient to ensure insulin treatment is adapted to patient needs and preferences.	Older people are offered more personalised care which fosters active patient engagement in self-care.	Upskill practice nurses to provide on-going support by tailoring guidance based on patient’s lifestyle and individual needs	Training (imparting skills)
Lack of confidence in handling insulin (Transition to insulin)	More support with self-monitoring of blood glucose and access to monitoring equipment to enhance safe insulin use.	Enhancing older people’s’ self-efficacy improves their ability to independently manage insulin.	Develop user-friendly insulin pens with clear large, easy to read dose indicators for older people with cataract or similar eye issues. Also, more suitable for individuals with limited dexterity.	Enablement (increasing means or reducing barriers to increase capability beyond environmental restructuring)
Patients aspire to assume greater authority over their own bodies–- a transition captured by the shift from ‘compliance’ to ‘concordance’ (move away from the patients adhering to medical instructions (‘compliance’) toward an active participation of patients in decisions (‘concordance’). (Transition to insulin, healthcare services)	Patients should be empowered to be involved in treatment decisions, and regularly asked: • Are you happy to continue with insulin? • Are you willing to continue? • What about the type of insulin?	Positive communication with older individuals enhances their understanding of the benefits of insulin, empowering them and improving their self-efficacy.	Healthcare professionals have positive communication with patients to explain the benefits of insulin.	Persuasion (using communication to induce positive or negative feelings or stimulate action
Lack of individualised information reflecting variability in learning styles, cognitive ability, cultural background, and information needs. (Transition to insulin, social challenges)	A range of different informational resources (e,g, Online educational tool, multi languages, text, audio or video options, ‘Bite size’ information) which people can access depending on their individual needs.	Tailored and age appropriate. Education for older individuals increases their understanding and, consequently, enhances their self-efficacy.	A website with the option to translate and no need for reading can listen to information, like audible as well as pictograms	Education (increase knowledge or understanding)
Lack of diet and lifestyle advice in relation to insulin. (Healthcare services)	Individualised diet advice with in-person sessions with a dietician supported with online education and self-help resources.	Customised and age-specific education for older individuals not only boosts their comprehension but also elevates their self-efficacy.	A diet website which offers personalised nutrition guidance, available anytime, anywhere. It caters to different learning styles with translation and auditory features, and visual aids enhance understanding for all users.	Education (increase knowledge or understanding)
Lack of access and continuity to general practitioner (GP). (Healthcare services)	Virtual/phone conversations with GP instead of face-to-face appointments to support insulin initiating and treatment	Older people are empowered to navigate the complexities of insulin administration and address concerns effectively.	Older people on insulin and carers have a dedicated access to practice nurse to ask questions	Restriction (using rules to reduce/increase the opportunity to engage in target behaviour)

### The logic model

**Fig 2:**
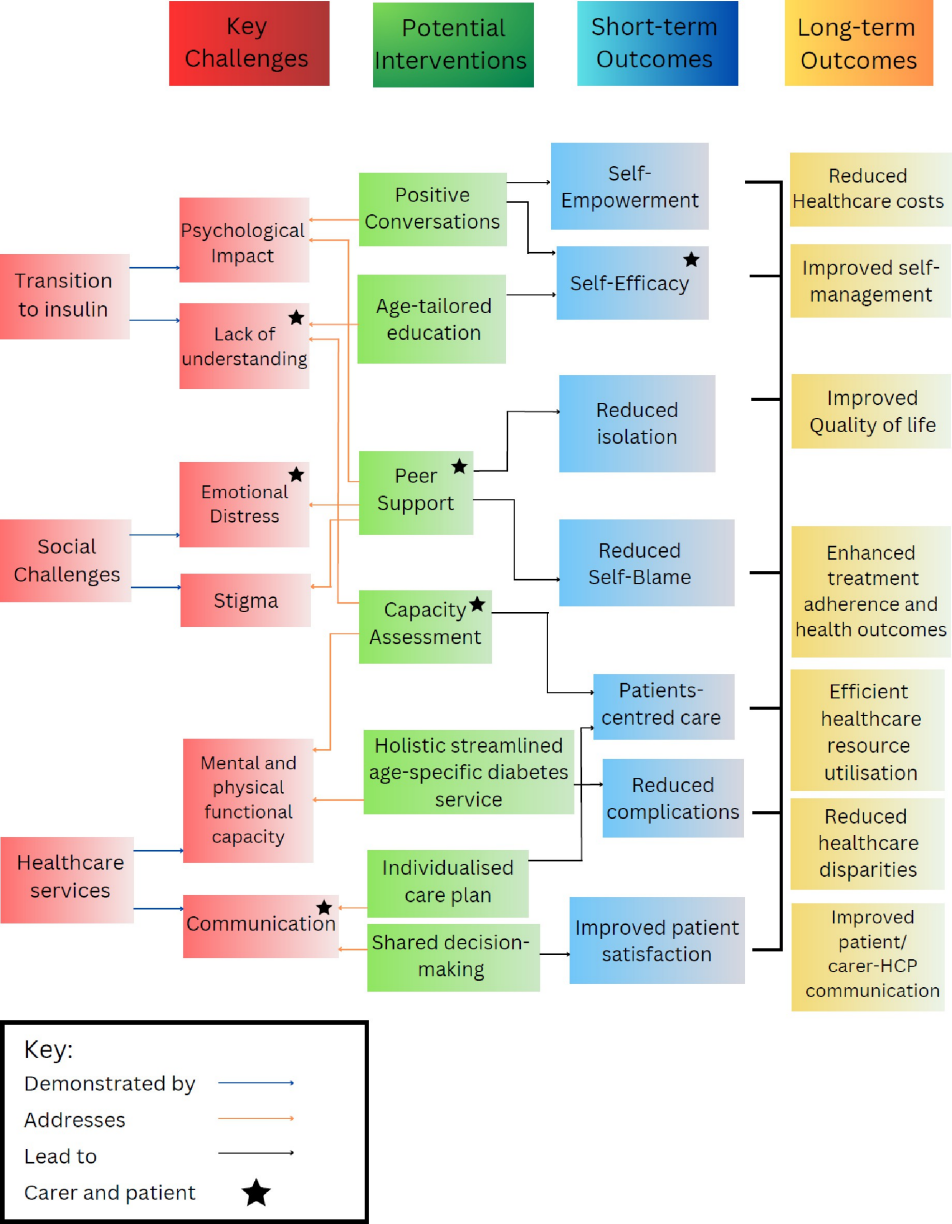
Logic model of proposed initiatives for improving older individuals’ experiences of insulin transition and management–- priorities, activities, outcomes and impacts.

This logic model (Fig 2) delineates a possible pathway for older people deemed suitable for insulin treatment, reflecting intervention solutions proposed by the participants at the co-design workshop. The main issues facing older people, which were derived from challenges identified in the interviews (Table 3) and were grouped into three main areas for the purposes of the logic model. These were: transition to insulin (encompassing aspects related to patient knowledge, readiness, and the psychological impact on both patients and caregivers); social challenges (including emotional support and addressing stigma); and healthcare services (with a specific focus on age-appropriate diabetes services. In the feedback focus groups, participants pinpointed specific target behaviours that needed modification. These included altering the approach to providing information to both carers and older individuals and actively involving older people in decision-making about their care. Subsequently, during these focus groups, consensus was reached on the identified target behaviours that necessitated change. In the subsequent co-design workshop, participants further aligned on the necessary modifications to these behaviours. To illustrate, to support older peoples’ transition to insulin, challenges included a psychological impact of moving on to insulin and a lack of knowledge about insulin management. A training intervention was proposed by participants to empower nurses to engage in positive and motivating conversations with patients. Similarly, an education intervention was proposed to enhance older peoples’ comprehension of insulin regimens which was tailored to their age and individual capacity. Both of these approaches were considered by stakeholders as having the potential to increase patient empowerment and self-efficacy. In the longer term, research suggests that such input may increase self-management and reduce healthcare costs.

## Discussion

This research uncovered the absence of a well-defined care process for older individuals undergoing insulin treatment. It is evident from stakeholder input that what is needed is a personalised care pathway that considers each individual’s unique requirements. We have chosen to guide our intervention development using the BCW. This decision is driven by our goal to encourage older people, family carers, and healthcare professionals to embrace new behaviours that will enhance their experiences with insulin transition and management [22].

Older people with T2DM emphasised that positive conversations with doctors and nurses significantly contributed to their initial acceptance of insulin [46] and encourages patients to accept and adhere to insulin therapy. Previous research has shown that social support from peers plays a crucial role when individuals are initiated on insulin therapy [47]. Peer support can promote more effective management behaviours and helps individuals cope with negative emotions [48]. This study highlights the importance of providing appropriate age-tailored education to enhance self-efficacy and self-management. A previous study [49] highlighted that patient knowledge and understanding of insulin treatment can significantly influence their chances of successful transition. Therefore, it is crucial to assess an older person’s understanding and provide access to education when needed. The importance of shared decision-making to fostering patient satisfaction with treatment is highlighted by Montro et al. [50].

Capacity assessments are essential to tailor care according to individual patient needs [51]. Sinclair et al. [52] emphasise the heterogeneity of the older population. Throughout their insulin journey, older individuals may experience frailty, requiring adaptations in their care to accommodate changes in both physical and mental capacity. Conducting capacity assessments is an ongoing necessity, not only at the initiation of insulin but also throughout the treatment process. Evaluating an older person’s ability to perform daily activities [53] which can influence their aptitude for administering insulin or monitoring glucose levels is important. Furthermore, assessing their visual capabilities is essential for tasks such as reading insulin labels, accurately measuring doses, and checking glucose levels. Evaluating fine motor skills is crucial, particularly for the precise administration of insulin using devices like pens or syringes. Additionally, understanding psychological capacity, including an older person’s motivation for self-management and their ability to remember when to take insulin, is integral to a comprehensive assessment [54].

Upskilling practice nurses represents a significant transformation in the current care paradigm for older individuals on insulin. An expanded skill set can enable nurses to provide crucial support, capacity assessments, education, and emotional assistance. There is certainly evidence of a positive impact of individualised care provided by nurses on patient outcomes [55]. Active efforts by some nurses to deliver personalised care, results in increased time spent with patients and higher patient’s satisfaction levels [56]. Furthermore, expanding nursing roles to encompass holistic care, extending beyond diabetes management, has been shown to have positive implications [57]. Nurses should be trained to have adequate knowledge about diabetes and insulin [58] to ensure sufficient consultation skills. While nurses play a crucial role, a team-based approach in diabetes care is vital for patient-centred care [59]. However, this approach may lead to work overload, especially for nurses who make take on the responsibilities of other healthcare providers. This challenge may be exacerbated by a shortage of manpower which is a persistent issue in diabetes care [60]. Additionally, nurses in our study reported feeling unprepared for advanced roles in diabetes care, which emphasises the ongoing need for capacity building to maximise the impact of their role.

### Future research

The proposed logic model highlights the need for healthcare professionals to change their behaviour alongside a structural alteration in care delivery to improve older peoples’ experiences of insulin initiation and management. This proposed strategy aims to assist people in enhancing their insulin usage in a personalised manner, with the goal of improving their experiences. Additionally, the pathway proposes optimising the support given by Practice Nurses (PNs) and General Practitioners (GPs). These proposed improvements are predicated on the positive impact they should have on both older peoples’ quality of life and overall health. We have identified the intervention functions (BCW) of these novel approaches to diabetes care in order to guide their development in behavioural terms. Further development of the personalised interventions described by stakeholders such as the “insulin passport” would require a collaborative design process involving older people, their caregivers, and healthcare providers. This would be crucial, especially within the context of general medical practice.

### Limitations

The evolving pandemic restrictions and guidelines had an impact on certain aspects of this co-design project. In the early stages of the COVID-19 pandemic, challenges with connectivity, digital access and digital literacy, particularly among older people and ethnic minorities, were reported [61]. Therefore, before resuming co-design workshops with older people, we assessed participant’’ technical capabilities and digital readiness. We ensured that those facing difficulties with mobile data allowances or whose current devices did not support virtual meetings were offered assistance. We provided clear instructions for downloading the MS Teams TM app. To ensure a smooth transition to virtual meetings, we organised a practice virtual session to test internet connectivity and so that participants could familiarise themselves with platform features like chat, raising hands, and adjusting camera settings. Additionally, this virtual practice provided an opportunity for us to connect with participants and develop rapport, which helped ease any initial discomfort. All participants were encouraged to send follow-up emails, especially if they encountered connectivity issues during the co-design workshop. Throughout the co-design process, we maintained open communication through emails, progress updates, and action points.

Initially, obtaining NHS ethics approval was delayed significantly due to COVID-related disruptions. Consequently, we opted to recruit participants from DUK groups and the NIHR people in research network. However, DUK groups suspended their meetings during the pandemic which further limited access to potential participants. Although the challenge of online recruitment made it difficult to maximise variation in the sample, recruiting from DUK groups across the UK rather than at local clinics ensured that older people from a wider range of geographical locations participated. Recruiting and retaining participants virtually also presented challenges. To accommodate participant withdrawals at various stages, we modified the EBCD approach, focusing on setup, feedback meetings, and a single co-design session. Despite these adaptations, the study yielded valuable and novel data about the experiences of older individuals with T2DM and their caregivers.

Whilst our original goal was to recruit a larger number of participants, we were able to gather substantial insights from the smaller sample recruited, allowing us to complete the EBCD process. The concept of ‘information power,’ highlights that when the sample holds a wealth of relevant information, a smaller participant group can still be sufficient for the study’s purposes [62]. While the limited sample size in this study may be viewed as a potential constraint, the in-depth dialogues conducted by an experienced researcher during interviews and focus groups greatly enriched the information quality.

Finally, we acknowledge that constructing a logic model involves an interpretive process. There could potentially be alternative models or theoretical frameworks that align with the program’s logic, and the model might have taken a different form if developed from a non-psychological perspective. Methodological robustness was achieved through constant reflection throughout the modelling process. This involved cross-referencing with co-authors’ interpretations and an ongoing iterative process to continually refine the "if-then" statements and the model diagram. Given only one joint co-design session was held, there was no opportunity for further iterative development of the logic model. Although some HCPs reviewed the logic model as part of the development process, additional feedback from older people would have also been useful.

## Conclusion

This EBCD study revealed behavioural changes which need to occur within health professionals, individuals with T2DM and their caregivers in order to improve older peoples’ experiences of insulin initiation and management. Three pivotal features emerged during the EBCD process: the transition to insulin, social challenges, and aspects related to healthcare services. These key features played a central role in shaping the insights and improvements derived from the EBCD approach. This study has proposed a logic model to target behavioural changes and future research will need to focus on assessing its feasibility and effectiveness at achieving improvements.

## Supporting information

S1 File ISSM COREQ checklist. (PDF)

## References

[1] Abdelhafiz A, Bisht S, Kovacevic I, Pennells D, Sinclair A: Insulin in Frail, Older People with Type 2 Diabetes—Low Threshold for Therapy. *Diabetology* 2022, 3(2):369–383.

[2] Bellary S, Kyrou I, Brown JE, Bailey CJ: Type 2 diabetes mellitus in older adults: clinical considerations and management. *Nature Reviews Endocrinology* 2021, 17(9):534–548. doi: 10.1038/s41574-021-00512-2 34172940

[3] Hassali MA, Ching M-W, Yusoff ZM, Hussein Z, Alrasheedy AA, Al-Tamimi SK, et al: ‘Why I do not want to take insulin shots’: Findings from a qualitative study among diabetic patients in Malaysia. *Journal of Public Health* 2014, 22:3–11.

[4] Soylar P, Kadioglu B, Kilic K: Investigation of the barriers about insulin therapy in patients with type 2 diabetes. *Nigerian Journal of Clinical Practice* 2020, 23(1):98–102. doi: 10.4103/njcp.njcp_138_19 31929214

[5] Bahrmann A, Abel A, Zeyfang A, Petrak F, Kubiak T, Hummel J, et al: Psychological insulin resistance in geriatric patients with diabetes mellitus. *Patient Education and Counseling* 2014, 94(3):417–422. doi: 10.1016/j.pec.2013.11.010 24341962

[6] Strain WD, Down S, Brown P, Puttanna A, Sinclair A: Diabetes and frailty: an expert consensus statement on the management of older adults with type 2 diabetes. *Diabetes Therapy* 2021, 12:1227–1247. 33830409 10.1007/s13300-021-01035-9 PMC8099963

[7] Wolffenbuttel BH, Drossaert CH, Visser AP: Determinants of injecting insulin in elderly patients with type II diabetes mellitus. *Patient Education and Counseling* 1993, 22(3):117–125. doi: 10.1016/0738-3991(93)90091-a 8153033

[8] Okazaki K, Shingaki T, Cai Z, Perez-Nieves M, Fisher L: Successful healthcare provider strategies to overcome psychological insulin resistance in Japanese patients with type 2 diabetes. *Diabetes Therapy* 2019, 10:1823–1834. doi: 10.1007/s13300-019-0664-9 31286432 PMC6778551

[9] Langerman C, Forbes A, Robert G: The experiences of insulin use among older people with Type 2 diabetes mellitus: A thematic synthesis. *Primary Care Diabetes* 2022. doi: 10.1016/j.pcd.2022.08.008 36089508

[10] Mulvale G, Robert G: Engaging vulnerable populations in the co-production of public services. In., vol. 44: Taylor & Francis; 2021: 711–714.

[11] Robert G, Locock L, Williams O, Cornwell J, Donetto S, Goodrich J: Co-producing and co-designing: Cambridge University Press; 2022.

[12] O’Cathain A, Croot L, Duncan E, Rousseau N, Sworn K, Turner KM, et al: Guidance on how to develop complex interventions to improve health and healthcare. *BMJ open* 2019, 9(8):e029954. doi: 10.1136/bmjopen-2019-029954 31420394 PMC6701588

[13] O’Cathain A, Croot L, Sworn K, Duncan E, Rousseau N, Turner K, et al: Taxonomy of approaches to developing interventions to improve health: a systematic methods overview. *Pilot and feasibility studies* 2019, 5(1):1–27. 30923626 10.1186/s40814-019-0425-6 PMC6419435

[14] Robert G, Cornwell J, Locock L, Purushotham A, Sturmey G, Gager M: Patients and staff as codesigners of healthcare services. *Bmj* 2015, 350. doi: 10.1136/bmj.g7714 25670179

[15] Bate P, Robert G: Bringing user experience to healthcare improvement: the concepts, methods and practices of experience-based design: Radcliffe publishing; 2007.

[16] Locock L, Robert G, Boaz A, Vougioukalou S, Shuldham C, Fielden J, et al: Using a national archive of patient experience narratives to promote local patient-centered quality improvement: an ethnographic process evaluation of ‘accelerated’experience-based co-design. *Journal of Health Services Research & Policy* 2014, 19(4):200–207. doi: 10.1177/1355819614531565 24840387

[17] Silcock J, Marques I, Olaniyan J, Raynor DK, Baxter H, Gray N, et al: Co‐designing an intervention to improve the process of deprescribing for older people living with frailty in the United Kingdom. *Health Expectations* 2023, 26(1):399–408. doi: 10.1111/hex.13669 36420768 PMC9854320

[18] Bowen S, McSeveny K, Lockley E, Wolstenholme D, Cobb M, Dearden A: How was it for you? Experiences of participatory design in the UK health service. *CoDesign* 2013, 9(4):230–246.

[19] Tsianakas V, Maben J, Wiseman T, Robert G, Richardson A, Madden P, et al: Using patients’ experiences to identify priorities for quality improvement in breast cancer care: patient narratives, surveys or both? *BMC health services research* 2012, 12:1–11. 22913525 10.1186/1472-6963-12-271 PMC3466127

[20] Donetto S, Pierri P, Tsianakas V, Robert G: Experience-based co-design and healthcare improvement: realizing participatory design in the public sector. *The Design Journal* 2015, 18(2):227–248.

[21] Green T, Bonner A, Teleni L, Bradford N, Purtell L, Douglas C, et al: Use and reporting of experience-based codesign studies in the healthcare setting: a systematic review. *BMJ quality & safety* 2020, 29(1):64–76. doi: 10.1136/bmjqs-2019-009570 31548278

[22] Fylan B, Tomlinson J, Raynor DK, Silcock J: Using experience-based co-design with patients, carers and healthcare professionals to develop theory-based interventions for safer medicines use. *Research in Social and Administrative Pharmacy* 2021, 17(12):2127–2135. doi: 10.1016/j.sapharm.2021.06.004 34187746

[23] Bradway M, Morris RL, Giordanengo A, Årsand E: How mHealth can facilitate collaboration in diabetes care: qualitative analysis of co-design workshops. *BMC health services research* 2020, 20(1):1–20. doi: 10.1186/s12913-020-05955-3 33256732 PMC7706243

[24] Michie S, Richardson M, Johnston M, Abraham C, Francis J, Hardeman W, et al: The behavior change technique taxonomy (v1) of 93 hierarchically clustered techniques: building an international consensus for the reporting of behavior change interventions. *Annals of behavioral medicine* 2013, 46(1):81–95. doi: 10.1007/s12160-013-9486-6 23512568

[25] Ebenso B, Manzano A, Uzochukwu B, Etiaba E, Huss R, Ensor T, et al: Dealing with context in logic model development: reflections from a realist evaluation of a community health worker programme in Nigeria. *Evaluation and Program Planning* 2019, 73:97–110. doi: 10.1016/j.evalprogplan.2018.12.002 30578941 PMC6403102

[26] Mills T, Lawton R, Sheard L: Advancing Complexity Theory in Health Services Research: The Logic of Logic Models. *BMC Medical Research Methodology* 2019, 19(55). 10.1186/s12874-019-0701-4 PMC6419426 30871474

[27] Skivington K, Matthews L, Simpson SA, Craig P, Baird J, Blazeby JM, et al: A new framework for developing and evaluating complex interventions: update of Medical Research Council guidance. *bmj* 2021, 374. doi: 10.1136/bmj.n2061 34593508 PMC8482308

[28] Bate P, Robert G: Experience-based design: from redesigning the system around the patient to co-designing services with the patient. *Quality & safety in health care* 2006, 15(5):307–310. doi: 10.1136/qshc.2005.016527 17074863 PMC2565809

[29] Dewar B, Mackay R, Smith S, Pullin S, Tocher R: Use of emotional touchpoints as a method of tapping into the experience of receiving compassionate care in a hospital setting. *Journal of Research in Nursing* 2010, 15(1):29–41.

[30] Gage M, Kolari P: Making emotional connections through participatory design. 2002. In.; 2018.

[31] Raynor DK, Ismail H, Blenkinsopp A, Fylan B, Armitage G, Silcock J: Experience‐based co‐design—adapting the method for a researcher‐initiated study in a multi‐site setting. *Health Expectations* 2020, 23(3):562–570. doi: 10.1111/hex.13028 32045087 PMC7321746

[32] Michie S, Van Stralen MM, West R: The behaviour change wheel: a new method for characterising and designing behaviour change interventions. *Implementation science* 2011, 6(1):1–12. doi: 10.1186/1748-5908-6-42 21513547 PMC3096582

[33] McAllister S, Simpson A, Tsianakas V, Canham N, De Meo V, Stone C, et al: Developing a theory-informed complex intervention to improve nurse–patient therapeutic engagement employing Experience-based Co-design and the Behaviour Change Wheel: an acute mental health ward case study. *BMJ open* 2021, 11(5):e047114. doi: 10.1136/bmjopen-2020-047114 33986066 PMC8126294

[34] Kwok BC, Wong WP, Remedios L: Improving centre-based group exercise participation of older adults using the behaviour change wheel. *BMJ Open Quality* 2021, 10(1):e001078. doi: 10.1136/bmjoq-2020-001078 33589505 PMC7887340

[35] EBCD toolkit [https://www.kingsfund.org.uk/projects/ebcd]

[36] Sinclair AJ, Paolisso G, Castro M, Bourdel-Marchasson I, Gadsby R, Mañas LR: European Diabetes Working Party for Older People 2011 clinical guidelines for type 2 diabetes mellitus. Executive summary. Diabetes & metabolism 2011, 37:S27–S38. 22183418 10.1016/S1262-3636(11)70962-4

[37] Ritchie J, Spencer L, Bryman A, Burgess RG: Analysing qualitative data. In.: Routledge, London; 1994.

[38] Lincoln YS, Guba EG: But is it rigorous? Trustworthiness and authenticity in naturalistic evaluation. New directions for program evaluation 1986, 1986(30):73–84.

[39] Papoulias C: Showing the unsayable: Participatory visual approaches and the constitution of ‘Patient Experience’in healthcare quality improvement. *Health Care Analysis* 2018, 26(2):171–188. doi: 10.1007/s10728-017-0349-3 29038985 PMC5899993

[40] Thoring K, Müller RM: Understanding design thinking: A process model based on method engineering. In: *DS 69*: *Proceedings of E&PDE 2011*, *the 13th International Conference on Engineering and Product Design Education*, *London*, *UK*, *08–0909 2011*: 2011; 2011: 493–498.

[41] Knapp J, Zeratsky J, Kowitz B: Sprint: How to solve big problems and test new ideas in just five days: Simon and Schuster; 2016.

[42] Jones L, Nabil S, Girouard A: Wearable Crazy Eights: Wearable Ideation Methods for Encouraging Divergent Design Concepts. In: *Proceedings of the Fifteenth International Conference on Tangible*, *Embedded*, *and Embodied Interaction*: 2021; 2021: 1–7.

[43] Kellogg W: Logic Model Development Guide. Kellogg Foundation. In.; 2004.

[44] Knowlton LW, Phillips CC: The logic model guidebook: Better strategies for great results: Sage; 2012.

[45] Tong A, Sainsbury P, Craig J: Consolidated criteria for reporting qualitative research (COREQ): a 32-item checklist for interviews and focus groups. *International journal for quality in health care* 2007, 19(6):349–357. doi: 10.1093/intqhc/mzm042 17872937

[46] Mathew BK, De Roza JG, Liu C, Goh LJ, Ooi CW, Chen E, et al: Which Aspect of Patient–Provider Relationship Affects Acceptance and Adherence of Insulin Therapy in Type 2 Diabetes Mellitus? A Qualitative Study in Primary Care. *Diabetes*, *metabolic syndrome and obesity*: *Targets and therapy* 2022:235–246. doi: 10.2147/DMSO.S344607 35153494 PMC8828446

[47] Warshaw H, Hodgson L, Heyman M, Oser TK, Walker HR, Deroze P, et al: The role and value of ongoing and peer support in diabetes care and education. *The Diabetes Educator* 2019, 45(6):569–579. doi: 10.1177/0145721719882007 31617467

[48] Fisher EB, Tang PY, Coufal M, Liu Y, Luu SL, Evans M, et al: Peer support. In: *Chronic illness care*: *Principles and practice*. edn.: Springer; 2023: 113–127.

[49] Polonsky WH, Arsenault J, Fisher L, Kushner P, Miller EM, Pearson TL, et al: Initiating insulin: how to help people with type 2 diabetes start and continue insulin successfully. *International journal of clinical practice* 2017, 71(8). doi: 10.1111/ijcp.12973 28735508 PMC5601201

[50] Montori VM, Gafni A, Charles C: A shared treatment decision‐making approach between patients with chronic conditions and their clinicians: the case of diabetes. *Health Expectations* 2006, 9(1):25–36. doi: 10.1111/j.1369-7625.2006.00359.x 16436159 PMC5060323

[51] Munshi MN, Slyne C, Segal AR, Saul N, Lyons C, Weinger K: Simplification of insulin regimen in older adults and risk of hypoglycemia. *JAMA Internal Medicine* 2016, 176(7):1023–1025. doi: 10.1001/jamainternmed.2016.2288 27273335

[52] Sinclair A, Dunning T, Rodriguez-Mañas L: Diabetes in older people: new insights and remaining challenges. *The lancet Diabetes & endocrinology* 2015, 3(4):275–285. doi: 10.1016/S2213-8587(14)70176-7 25466523

[53] Vicente MC, Silva CRRd, Pimenta CJL, Bezerra TA, Lucena HKVd, Valdevino SC, et al: Functional capacity and self-care in older adults with diabetes mellitus. *Aquichan* 2020, 20(3).

[54] Tanqueiro M: Self-care management in older people with diabetes: systematic review of literature. *Referência* 2013, 3(9):151–160.

[55] Li D, Elliott T, Klein G, Ur E, Tang TS: Diabetes nurse case management in a Canadian tertiary care setting: Results of a randomized controlled trial. *Canadian journal of diabetes* 2017, 41(3):297–304. doi: 10.1016/j.jcjd.2016.10.012 28318938

[56] Doran D, Harrison MB, Laschinger H, Hirdes J, Rukholm E, Sidani S, et al: Relationship between nursing interventions and outcome achievement in acute care settings. Research in nursing & health 2006, 29(1):61–70. doi: 10.1002/nur.20110 16404735

[57] Stenner KL, Courtenay M, Carey N: Consultations between nurse prescribers and patients with diabetes in primary care: A qualitative study of patient views. *International journal of nursing studies* 2011, 48(1):37–46. doi: 10.1016/j.ijnurstu.2010.06.006 20627198

[58] Jansink R, Braspenning J, van der Weijden T, Elwyn G, Grol R: Primary care nurses struggle with lifestyle counseling in diabetes care: a qualitative analysis. *BMC family practice* 2010, 11:1–7. 20500841 10.1186/1471-2296-11-41 PMC2889883

[59] Alshammari M, Windle R, Bowskill D, Adams G: The role of nurses in diabetes care: a qualitative study. *Open Journal of Nursing* 2021, 11(8):682–695.

[60] Beck J, Greenwood DA, Blanton L, Bollinger ST, Butcher MK, Condon JE, et al: 2017 National standards for diabetes self-management education and support. *The Diabetes Educator* 2018, 44(1):35–50. 10.1177/0145721718754797 29346744

[61] Litchfield I, Shukla D, Greenfield S: Impact of COVID-19 on the digital divide: a rapid review. *BMJ open* 2021, 11(10):e053440. doi: 10.1136/bmjopen-2021-053440 34642200 PMC8520586

[62] Malterud K, Siersma V, Guassora AD: Information power: Sample content and size in qualitative studies. 2021. 10.1177/1049732315617444 26613970

